# TPX2 expression as a negative predictor of gemcitabine efficacy in pancreatic cancer

**DOI:** 10.1038/s41416-023-02295-x

**Published:** 2023-05-04

**Authors:** Michael Guenther, Sai Agash Surendran, Michael Haas, Volker Heinemann, Michael von Bergwelt-Baildon, Jutta Engel, Jens Werner, Stefan Boeck, Steffen Ormanns

**Affiliations:** 1 Institute of Pathology, Faculty of Medicine, Ludwig-Maximilians-University, Munich, Germany; 2 German Cancer Consortium (DKTK), Partner Site, Munich, Germany; 3 Department of Internal Medicine III, Grosshadern University Hospital, Ludwig-Maximilians-University, Munich, Germany; 4 Munich Cancer Registry (MCR), Munich Tumor Centre (TZM), Institute for Medical Information Processing, Biometry and Epidemiology, Ludwig-Maximilians-University, Munich, Germany; 5 Department of General, Visceral and Transplant Surgery, Ludwig-Maximilians-University, Munich, Germany

**Keywords:** Predictive markers, Tumour biomarkers, Pancreatic cancer

## Abstract

**Background:**

Targeting protein for Xenopus kinesin-like protein 2 (TPX2) overexpression in human tumours is associated with increased malignancy. Its effect on gemcitabine resistance in pancreatic ductal adenocarcinoma (PDAC) has not been studied yet.

**Methods:**

The prognostic impact of TPX2 expression was examined in the tumour tissue of 139 patients with advanced PDAC (aPDAC) treated within the AIO-PK0104 trial or translational trials and of 400 resected PDAC (rPDAC) patients. The findings were validated using RNAseq data of 149 resected PDAC patients.

**Results:**

In the aPDAC cohorts, 13.7% of all samples showed high TPX2 expression, conferring significantly shorter progression-free survival (PFS, HR 5.25, *P* < 0.001) and overall survival times (OS, HR 4.36, *P* < 0.001) restricted to gemcitabine-based treated patients (*n* = 99). In the rPDAC cohort, 14.5% of all samples showed high TPX2 expression, conferring significantly shorter disease-free survival times (DFS, HR 2.56, *P* < 0.001) and OS times (HR 1.56, *P* = 0.04) restricted to patients treated with adjuvant gemcitabine. RNAseq data from the validation cohort confirmed the findings.

**Conclusions:**

High TPX2 expression may serve as a negative predictor of gemcitabine-based palliative and adjuvant chemotherapy in PDAC and could be used to inform clinical therapy decisions.

**Clinical trial registry:**

The clinical trial registry identifier is NCT00440167.

## Background

Despite decades of basic and translational research pancreatic ductal adenocarcinoma (PDAC) remains one of the epithelial neoplasms with the poorest prognosis [1]. Even after potentially curative resection, 5-year survival rates are about 20%, whereas in the metastatic disease, which accounts for 80% of all cases, median survival times range between 6.7 and 11.1 months, depending on the applied palliative therapy [2]. In the operable situation, the introduction of routine adjuvant chemotherapy increased disease-free survival times (DFS) and overall survival times (OS) significantly; first established with adjuvant gemcitabine [3] which has been partially replaced by more active regimens like FOLFIRINOX only in clinically fit patients more recently [4]. In the metastatic, or locally advanced situation, palliative chemotherapy is nearly the only therapy option left, in which FOLFIRINOX as the most active regimen offers the longest survival times at the cost of high toxicities [5], precluding this regimen for the many frail patients in routine practice. However, in both clinical situations, a biomarker-based personalised medicine approach may help to select the right therapy for the right patient to improve outcomes and reduce side effects.

Targeting protein for Xenopus kinesin-like protein 2 (TPX2) is one of the four Aurora cofactors and plays a pivotal role in the formation of microtubules of the spindles in the process of mitosis [6]. Thus its expression is strongest in the G1 and S phase of the cell cycle [7]. Its increased expression may promote malignant transformation by amplification of centrosomes, failures in chromosome segregation and induction of DNA polyploidy [8]. Overexpression of TPX2 correlates with malignancy-associated disease characteristics such as disease stage, lymph node metastasis, vascular invasion and distant metastasis [9]. Thus, its overexpression has been associated with poor patient prognosis in several human malignancies, such as gastric cancer, colorectal cancer, oesophageal cancer, non-small cell lung cancer, prostate cancer and hepatocellular carcinoma [10]. Limited preclinical data on the role of TPX2 in PDAC showed its upregulation compared to normal tissue in this entity and suggested a potential function as therapy target [11, 12]. However, its prognostic implications have only been studied in publicly available small expression datasets on resected PDAC to date, without considering the potential effect of the applied adjuvant or palliative systemic therapies on patient outcome [12]. In this study, we examined the expression of TPX2 in PDAC tissue and determined its prognostic impact in large cohorts of resected and advanced PDAC patients with respect to the applied adjuvant or palliative treatment modalities and confirmed our findings in gene expression datasets of a large independent patient cohort.

## Materials and methods

### Patients and tumour material

Histologically confirmed formalin-fixed paraffin-embedded (FFPE) tumour tissue of PDAC patients irrespective of its origin (primary tumour, metastatic tissue) was retrieved from the archives of the pathology laboratories where the diagnosis of PDAC was first established. Patients’ overall survival (OS) was calculated from surgery or start of palliative chemotherapy to death by disease. Disease-free survival (DFS) was calculated from surgery to clinically or radiologically apparent disease relapse. Progression-free survival (PFS) was calculated from the start of palliative chemotherapy to the occurrence of disease progression. Advanced PDAC patients gave written informed consent to the use of their tumour material and clinical data upon study enrollment. The use of patient material and data of the resected PDAC cohort was approved by the ethics committee of the medical faculty LMU (project 20-081 to SO). The cohort comprised PDAC tissue of patients older than 18 years, resected between 2000 and 2016 with histologically confirmed PDAC, excluding all other pancreatic malignant entities as well as all patients deceased due to perioperative mortality within 30 days post surgery.

### Immunohistochemistry

A tissue microarray (TMA), comprising three cores of tumour tissue, one millimetre in diameter each, was constructed using a semi-automatic tissue arrayer (Beecher Instruments, CA, USA). Tumour tissue integrity on the TMA and tumour content was confirmed using routine hematoxyline-eosin staining by two pathologists (MG, SO). TPX2 expression was detected on 4-µm-thick sections by immunohistochemistry on a Ventana Benchmark Ultra autostainer instrument (Ventana, Tucson, AZ, USA) using an anti-TPX2 polyclonal rabbit antibody (HPA005487, Atlas antibodies, Bromma, Sweden) at a 1:100 dilution for 32 min, after incubation with cell conditioning reagent 1 (CC1, Ventana) for 64 min. The signal was detected using OptiView kits (Ventana). Appropriate positive controls (human normal tonsil tissue, Supplementary Fig. S1) were included in each staining run. The expression pattern and expression strength were evaluated by two pathologists (MG, SO) blinded to the patient outcome and tumours were classified as follows: TPX2 high: strong and continuous nuclear expression in most tumour cells; TPX2 low: moderate or weak nuclear expression in some tumour cells or absent signal. Tumours were scored independently, and discrepant cases were discussed until agreement was reached. Microfotographs were acquired on a camera-equipped Zeiss Axiovision microscope (Zeiss, Wetzlar, Germany) at 200-fold magnification using proprietary Zeiss software.

### Statistics and in silico analyses

Kaplan–Meier curves, Cox regression analyses and cross-tabulations were calculated using SPSS software (IBM, Ehningen, Germany) considering a *P* value of lower than 0.05 as statistically significant. Publicly available gene expression data from two independent PDAC cohorts [13, 14] were analysed using the online platform *Survexpress* [15]. Normalised RNAseq expression data was downloaded from the data repository of The Cancer Genome Atlas (TCGA firehose, https://www.cancer.gov/tcga). Corresponding clinical patient information was downloaded from Broad GDAC Firehose and NCI Genomic Data Commons (GDC Data Release v29.0) [16]. Propensity-score matching was conducted using pymatch (https://github.com/benmiroglio/pymatch) for Python (Anaconda Inc., Austin, TX, USA). TPX2 gene methylation data and comparison to normal pancreatic tissue were obtained using the DiseaseMeth 2.0 database [17]. A TPX2-associated gemcitabine-resistance score and the association of TPX2 expression with the expression of gemcitabine-resistance associated genes was calculated as described previously in the R statistical environment [18]. Optimal cutpoints defining low and high TPX2 expression were determined using maximally selected rank statistics (R package: MaxStat v. 0.7-25). Expression heatmaps were generated using heatmaply [19].

## Results

### High TPX2 expression correlates with poor outcome in advanced pancreatic cancer patients treated with gemcitabine-based chemotherapy

To examine a potential role of TPX2 in PDAC, we first interrogated two previously published independent expression datasets [13, 14] (*n* = 291 in total) and found a strong negative impact of high TPX2 expression on patients´ survival (Supplementary Fis. S2A, B). Moreover, PDAC tissue showed significantly decreased TPX2 gene methylation levels compared to normal pancreatic tissue (Supplementary Fig. S2C), as a potential reason for its overexpression. In addition, we did not detect significant TPX2 expression levels in non-neoplastic tissue adjacent to invasive PDAC, like exocrine parenchyma, exocrine pancreas with reactive changes and initial chronic pancreatitis, chronic pancreatitis, fat tissue or neural tissue (Supplementary Fig. S3A–H).

### Patient characteristics in the advanced PDAC cohort

To further explore the role of TPX2 in advanced PDAC, we examined the tumour tissue of 139 patients from two independent study cohorts for its expression by immunohistochemistry and assessed its correlation with the patients´ clinicopathological characteristics including outcome. Seventy-one patients were treated with erlotinib-containing palliative chemotherapy within the AIO-PK0104 trial [20]; 68 patients were treated with erlotinib-free chemotherapy within translational biomarker trials [21]. Seventy-nine patients were male, 60 were female. Median patient age was 62.5 years. Median follow-up was 19.4 months (95% CI 12.2–26.6) (95% CI 18.9–45.9) for PFS and 32.4 months for OS. Fifty-one patients had a Karnofsky performance score (KPS) of lower than or equal to 80 (Table 1). Most of the patients (*n* = 80) had poorly differentiated tumours (grade G3 or G4) which was significantly associated to inferior patient outcome (Supplementary Table S4). Ninety-nine patients received a gemcitabine-based 1st-line palliative chemotherapy, whereas 40 received a non-gemcitabine-based 1st-line regimen (capecitabine for the 30 patients from the AIO-PK0104 study cohort and 5-fluorouracil or other agents for the ten patients from the translational trials cohort), which conferred significantly shorter OS and PFS times (Table 1 and Supplementary Table S4).

**Table 1:** Comparison of clinicopathological patient characteristics as well as TPX2 expression between the non-gemcitabine-based and the gemcitabine-based palliative treatment subgroups in the advanced PDAC cohorts.

	Treatment arm, no (%)		
	Non-gemcitabine-based (*n* = 40)	Gemcitabine-based (*n* = 99)	*P* value (*χ*^2^)
Sex			
Female	22 (55.0)	57 (57.6)	0.78
Male	18 (45.0)	42 (42.4)	
Age (years)			
≤60	16 (40.0)	41 (41.4)	0.88
>60	24 (60.0)	58 (58.6)	
TPX2 expression			
Low	36 (90.0)	84 (84.8)	0.42
High	4 (10.0)	15 (15.2)	
KPS			
≤80	18 (45.0)	33 (14.9)	0.21
>80	22 (55.0)	65 (85.1)	
Grade group			
G1–G2	14 (37.5)	47 (21.2)	0.21
G3–G4	26 (62.5)	52 (78.8)	
Stage at therapy start			
Locally advanced	7 (17.5)	17 (17.2)	0.96
Metastatic	33 (82.5)	82 (82.8)	

### TPX2 expression in the advanced PDAC cohort

**Fig. 1:**
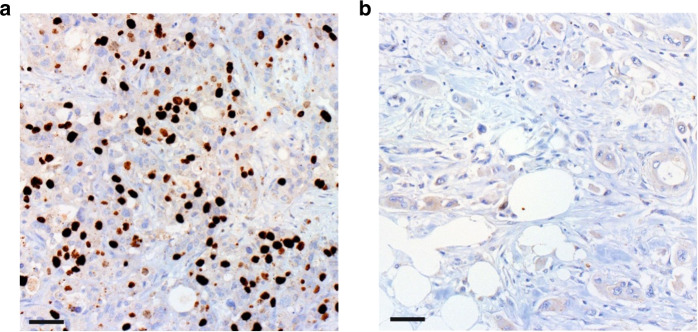
Differential expression of TPX2 in pancreatic cancer. Immunohistochemical detection of TPX2 expression in exemplary PDAC cases showing (**a**) high TPX2 expression and (**b**) low TPX2 expression. 200-fold magnification. Scale bars indicate 50 µm.

**Fig. 2:**
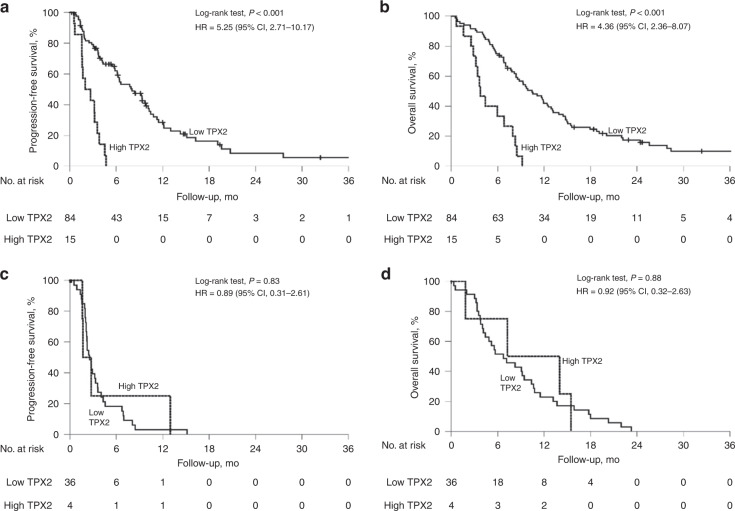
High TPX2 expression is associated with dismal prognosis in 1st-line gemcitabine-treated pancreatic cancer patients. Univariate analyses (Kaplan–Meier curves and log-rank tests) for PFS and OS in the gemcitabine 1st-line chemotherapy subgroup (**a**, **b**) as well as in the non-gemcitabine 1st-line chemotherapy subgroup (**c**, **d**), according to TPX2 expression. Crossed lines indicate censored cases.

We detected high TPX2 expression in 13.7% of all tumour samples (Fig. 1), which was significantly associated with shorter patient PFS (5.9 vs 2.0 months, *P* < 0.001, HR 2.74, 95% CI 1.63–4.61, Supplementary Table S5) and OS (9.3 vs 4.4 months, *P* < 0.001, HR 2.52, 95% CI 1.52–4.16, Supplementary Table S5). On subgroup analyses, high TPX2 expression retained its strong negative prognostic impact on PFS and OS in the patients treated with 1st-line gemcitabine-based palliative chemotherapy (PFS 8.1 vs 2.0 months, HR 5.25, 95% CI 2.71–10.17, *P* < 0.001; OS 10.5 vs 3.8 months, HR 4.36, 95% CI 2.36–8.07, *P* < 0.001; Fig. 2a, b), whereas no significant differences were detected in the patients treated with non-gemcitabine-based 1st-line regimens (PFS 2.5 vs 1.7 months, HR 0.89, 95% CI 0.31–2.61, *P* = 0.83, OS 6.7 vs 7.3, HR 0.92, 95% CI 0.32–2.63, *P* = 0.88; Fig. 2c, d). A separate analysis of both cohorts confirmed the findings from the entire cohort: high TPX2 expression correlated significantly with shorter PFS and OS in patients which received 1st-line gemcitabine-based treatment but not in non-gemcitabine 1st-line treated patients in the AIO-PK0104 study and the translational trials cohort (Supplementary Table S5).

In Cox multivariate regression analyses adjusting for tumour differentiation grade, KPS and type of 1st-line chemotherapy (where appropriate), we identified high TPX2 expression as independent negative prognostic tissue biomarker for PFS and OS in the 1st-line gemcitabine-treated patient subgroup (*P* < 0.001 each, Supplementary Table S6) but not in the non-gemcitabine 1st-line treatment subgroup as expected. Using cross-tabulations, we did not detect associations between high TPX2 expression and gender, age group, KPS, tumour grade nor type of 1st-line chemotherapy (Supplementary Table S7).

### High TPX2 expression is associated with poor prognosis in resected pancreatic cancer patients treated with adjuvant gemcitabine-based chemotherapy and correlates with gemcitabine resistance

**Fig. 3:**
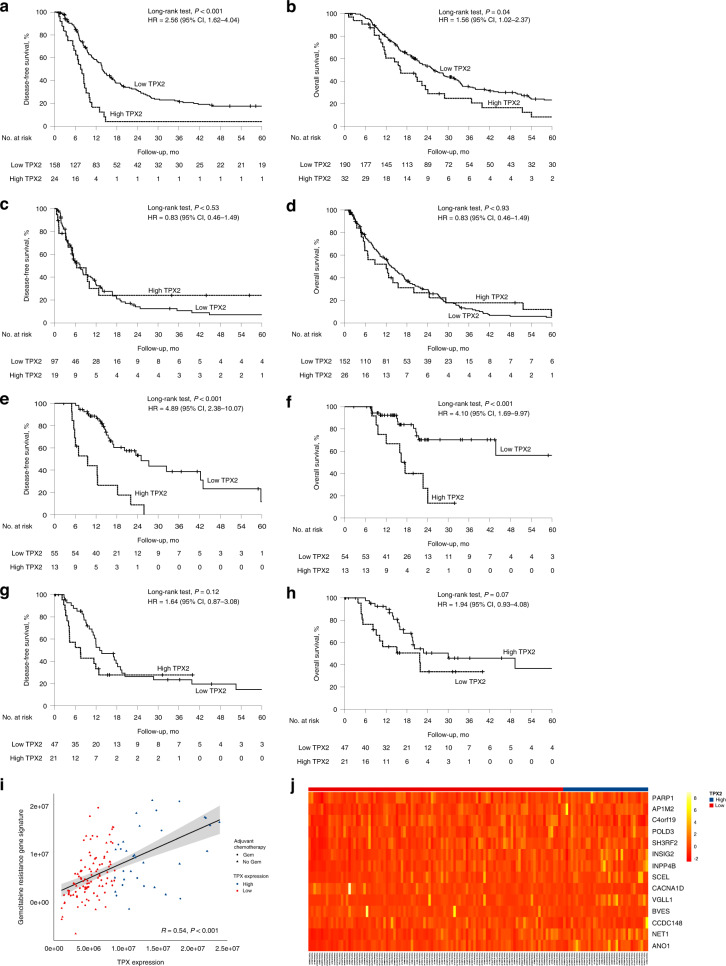
High TPX2 expression is associated with dismal prognosis in resected pancreatic cancer patients treated with adjuvant gemcitabine-based chemotherapy. Univariate analyses (Kaplan–Meier curves and log-rank tests) for DFS and OS in the gemcitabine-based adjuvant treatment cohort (**a**, **b**) and the non-gemcitabine-based adjuvant treatment cohort (**c**, **d**) according to TPX2 expression, as well as in the gemcitabine-based treated (**e**, **f**) and the non-gemcitabine-based treated (**g**, **h**) validation cohorts. Crossed lines indicate censored cases. Association of TPX2 expression and 14 gene-based gemcitabine-resistance expression signature (**i**). Correlation of TPX2 expression and the expression of gemcitabine-resistance-associated genes (**j**).

As we detected a significant impact of TPX2 expression on outcome limited to gemcitabine-based treated patients with advanced PDAC, we examined whether similar effects existed in resected PDAC, in which gemcitabine is still a widely employed regimen for adjuvant treatment. Thus, we examined TPX2 expression in the tumour tissue of 400 resected PDAC patients and tested its effect on patient outcome according to the type of adjuvant treatment they received. The study cohort consisted of 204 women and 196 men (median age 68 years, range 22–87 years), of which 222 received gemcitabine-based adjuvant treatment (aG) and 178 received either non-gemcitabine-based or no adjuvant treatment (naG, Table 2). We detected high TPX2 expression at similar rates in both cohorts (aG 14.4%, naG 14.6%, *P* = 0.96, Table 2), which conferred significantly shorter DFS and OS times in the aGC cohort (DFS 7.6 vs 13.8 months, HR 2.56, 95% CI 1.62–4.04, *P* < 0.001; OS 16.2 vs 25.8 months, HR 1.56, 95% CI 1.02–2.37, *P* = 0.04, Fig. 3a, b). However, in the naG cohort, high TPX2 expression did not affect outcome (DFS 6.4 vs 7.4 months, HR 0.83, 95% CI 0.46–1.49, *P* = 0.53; OS 12.0 vs 13.4 months, HR 0.98, 95% CI 0.62–1.54, *P* = 0.93, Fig. 3c, d). Cox multivariate regression analyses adjusting for differentiation grade, age, disease stage and R-status confirmed TPX2 expression as an independent prognosticator in the aG cohort (DFS *P* < 0.001, OS *P* = 0.01, Supplementary Table S8). As there existed significant differences in some clinicopathological parameters such as age, UICC stage and R-status between both cohorts (Table 2), we employed a propensity-score matching approach to eliminate these imbalances. In the resulting, well-balanced cohort, consisting of 95 patients in each group (Supplementary Table S9), we clearly confirmed the findings from the unmatched cohorts (Supplementary Fig. S10A–D).

**Table 2:** Comparison of clinicopathological patient characteristics as well as TPX2 expression between the non-gemcitabine-based and the gemcitabine-based adjuvant treatment subgroups in the resected PDAC cohort.

	Treatment arm, no (%)		
	Non-gemcitabine-based (*n* = 178)	Gemcitabine-based (*n* = 222)	*P* value (*χ*^2^)
Sex			
Female	92 (51.7)	112 (50.5)	0.81
Male	86 (48.3)	110 (49.5)	
Age (years)			
≤68	82 (46.1)	123 (55.4)	0.06
>68	96 (53.9)	99 (44.6)	
TPX2 expression			
Low	152 (85.4)	190 (85.6)	0.96
High	26 (14.6)	32 (14.4)	
UICC stage (2017)			
Stage IA	13 (7.3)	11 (5.0)	<0.001
Stage IB	32 (17.9)	50 (22.5)	
Stage IIA	19 (10.8)	15 (6.8)	
Stage IIB	48 (26.9)	85 (38.2)	
Stage III	31 (17.4)	50 (22.5)	
Stage IV	35 (19.7)	11 (5.0)	
pT (2017)			
pT1a	3 (1.7)	0 (0.0)	0.18
pT1b	2 (1.1)	3 (1.4)	
pT1c	18 (10.1)	26 (11.7)	
pT2	99 (55.6)	139 (62.6)	
pT3	53 (29.8)	53 (23.9)	
pT4	3 (1.7)	1 (0.4)	
pN (2017)			
pN0	77 (43.3)	81 (36.5)	0.15
pN1	54 (30.3)	88 (39.6)	
pN2	47 (26.4)	53 (23.9)	
R-status			
0	99 (55.6)	168 (75.7)	<0.001
1	79 (44.4)	54 (24.3)	
Grade group			
G1–G2	49 (27.5)	67 (30.2)	0.56
G3–G4	129 (72.5)	155 (69.8)

### TPX2 expression in the validation dataset

To further verify our data, we employed expression data from TCGA firehose, comprising 149 patients with available data on DFS as a validation cohort. Sixty-eight patients received gemcitabine-based adjuvant therapy (aG); 81 received either none or no gemcitabine-based adjuvant treatment (naG, Table 3). Importantly, high TPX2 mRNA levels within the patients´ tumour tissue conferred significantly decreased DFS and OS times in the aG patients (DFS 9.6 vs 25.1 months, HR 4.89, 95% CI 2.38–10.07, *P* < 0.001; OS 16.4 vs 66.9 months, HR 4.10, 95% CI 1.69–9.97, *P* < 0.001, Fig. 3e, f), but not in the naG patients (DFS 7.5 vs 13.0 months, HR 1.64, 95% CI 0.37–3.08, *P* = 0.12; OS 21.7 vs 30.0 months, HR 1.94, 95% CI 0.93–4.08, *P* = 0.07, Fig. 3g, h). To examine whether TPX2 expression was directly linked to gemcitabine resistance, we calculated a gemcitabine-resistance score based on a 14 gene expression signature. Tumours showing high TPX2 expression levels displayed high gemcitabine-resistance scores (Fig. 3i), as high TPX2 expression co-segregated with the expression of genes known to be associated with gemcitabine resistance [18] (Fig. 3j). Thus, in the TCGA firehose legacy dataset [22], TPX2 expression positively correlated with the expression of genes associated with gemcitabine resistance, such as ribonucleoside-diphosphate reductase large subunit (RRM1) [23] and polo-like-kinase-1 (PLK1) [24], but also correlated inversely with the expression of dipeptidase 1 (DPEP1), a gene previously shown to increase gemcitabine sensitivity in vitro [25] (Supplementary Fig. S11).

**Table 3:** Comparison of clinicopathological patient characteristics as well as TPX2 expression between the non-gemcitabine-based and the gemcitabine-based adjuvant treatment subgroups in the resected PDAC validation cohort.

	Treatment arm, no (%)		
	Non-gemcitabine-based (*n* = 81)	Gemcitabine-based (*n* = 68)	*P* value (*χ*^2^)
Sex			
Female	40 (49.4)	29 (42.6)	0.41
Male	41 (50.6)	39 (57.4)	
Age (years)			
≤68	41 (50.6)	34 (50.0)	0.94
>68	40 (49.4)	34 (50.0)	
TPX2 expression			
Low	46 (56.8)	49 (72.0)	0.05
High	35 (43.2)	19 (28.0)	
UICC stage (2017)			
Stage IA	0 (0)	5 (7.4)	0.16
Stage IB	10 (12.3)	8 (11.8)	
Stage IIA	5 (6.2)	5 (7.4)	
Stage IIB	31 (38.3)	30 (44.1)	
Stage III	31 (38.3)	18 (26.5)	
Stage IV	2 (2.5)	2 (3.0)	
pT (2017)			
pT1	1 (1.2)	6 (8.8)	0.13
pT2	45 (55.6)	39 (57.4)	
pT3	26 (32.1)	16 (23.5)	
pT4	2 (2.5)	1 (1.5)	
pN (2017)			
pN0	19 (23.5)	19 (28.0)	0.27
pN1	31 (38.3)	32 (47.0)	
pN2	30 (37.4)	17 (25.0)	
R-status			
0	38 (47.0)	45 (66.2)	0.09
1	32 (39.5)	18 (26.5)	
2	3 (3.7)	1 (1.5)	
Grade group			
G1–G2	61 (75.3)	44 (64.7)	0.16
G3–G4	20 (24.7)	24 (35.3)	

## Discussion

In contrast to other solid malignancies like non-small-cell lung cancer or breast cancer, in which predictive biomarkers entered routine clinical practice, to date none have been established for PDAC, a notoriously therapy-resistant disease. Thus, the characterisation of novel potential predictive biomarkers, verified in large independent PDAC cohorts is warranted and could inform therapy decisions on a molecular basis. A potential biomarker in this context is TPX2, which has previously been associated to inferior outcome in several solid malignancies [10]. Interestingly, although studied as prognostic biomarker in small cohorts of resected PDAC [12], the impact of TPX2 expression on outcome in advanced PDAC and on the response to gemcitabine, a commonly employed chemotherapeutic drug in the adjuvant situation, has not been thoroughly examined to date. In the present analysis, using well-curated datasets from defined large study cohorts comprising 688 patients overall, we show that high TPX2 expression is a biomarker associated with inferior outcome confined to advanced PDAC patients treated with gemcitabine-based palliative regimens and resected PDAC patients treated with gemcitabine-based adjuvant chemotherapy. Thus, we reason that TPX2 serves as a negative predictive biomarker for gemcitabine efficacy in both the palliative and the adjuvant situation. In line with our findings, high TXP2 expression co-segregates with the expression of genes associated with gemcitabine resistance, which results in a highly significant correlation of TPX2 expression and a previously described gemcitabine-resistance score [18].

### Molecular background of TPX2-mediated gemcitabine resistance

The exact molecular mechanism how TPX2 confers gemcitabine resistance remains elusive to date. After cellular uptake, gemcitabine is activated in the cytoplasm and inhibits DNA synthesis by incorporation in the DNA strand as “faulty” base, which leads to chain termination and induces replication stress [26]. One potential mechanism by which TPX2 may mediate gemcitabine resistance is through its control over aurora kinase A (AURKA) [8], which is strongly linked to Polo-like-kinase-1 (PLK1) activity [27, 28]. PLK1 regulates the activity of the protein origin recognition complex subunit 2 (ORC2)—which maintains DNA replication under gemcitabine treatment—and the activity of the HBO1 acetyltransferase complex—which activates cFOS and increases the expression of its target multidrug-resistance-protein MDR1-, both resulting in gemcitabine resistance [24]. Another potential mechanism of gemcitabine resistance is via TPX2-mediated repression of the Ser139-phosphorylated histone variant H2AX (γ-H2AX). Gemcitabine induces DNA damage through stalled replication forks and partial DNA chain termination, which results in nuclear γ-H2AX accumulation, inhibition of DNA synthesis, S-phase accumulation and activation of the S-phase checkpoint pathway, which ultimately leads to a halt in replication and consequently blocked proliferation [29]. Instead, TPX2 overexpression reduces γ-H2AX levels, thus inhibiting the checkpoint arrest and inhibition of DNA synthesis during gemcitabine treatment [30]. Conversely, TPX2 together with AURKA was recently shown to protect replication forks during replication stress [31]. In summary, we propose three potential mechanisms by which TPX2 mediates gemcitabine resistance: maintaining DNA replication, increased cellular drug export and inhibition of the checkpoint arrest pathway. However, considering the broad evidence on molecular mechanisms of gemcitabine resistance and the multiple cellular functions of TPX2, its involvement is likely multifactorial.

### Clinical implications

Although gemcitabine monotherapy has been largely replaced by more efficient chemotherapy regimens in the palliative situation, it remains an option for many patients whose performance status or comorbidities preclude combination chemotherapy [2, 32]. In fact, recent real-world data indicate that gemcitabine-based chemotherapy is still largely employed in advanced PDAC, according to which 23.2% of the patients received gemcitabine monotherapy and 41.7% were treated with gemcitabine-nab-paclitaxel [33]. The use of the latter regimen, although more efficient than gemcitabine monotherapy, is probably discouraged in tumours with high TPX2 expression, as it also confers resistance to paclitaxel therapy, at least in vitro [11]. Our findings on the effect of TPX2 on the efficacy of adjuvant gemcitabine, as measured by its impact on DFS however, may have direct implications for clinical routine practice, as adjuvant gemcitabine monotherapy is still widely in use to date.

### Limitations and strengths of the study—outlook

Our study has some limitations, like its retrospective nature for instance and the fact that neither the advanced PDAC study cohorts nor the resected PDAC cohort contain a FOLFIRINOX-treated patient subgroup, which currently is standard-of-care for clinically fit patients in both situations [4, 5]. Whereas the patients from the resected PDAC study cohort were all operated in a single large tertiary care academic centre, the patients in the advanced PDAC cohorts, however, were treated within randomised multicentric clinical trials [20]. Moreover, we used a propensity-score matching approach to overcome potential bias and additionally confirmed our findings in a well-curated validation cohort using RNA-based expression data. Importantly, the findings from both advanced PDAC cohorts perfectly match the findings in the resected patients, further strengthening our conclusion that the detection of high TPX2 expression may, in addition to the clinical situation of the patient, inform biomarker-based therapy decisions. Interestingly, with the advent of mRNA-based vaccination therapies, TPX2 itself was identified as a potential therapy target in PDAC [34]. Further research is necessary to elucidate the impact of TPX2 on currently available therapy regimens like FOLFIRINOX, and a validation of our findings in randomised control trials is highly desirable.

## Supplementary information

Figure S1

Figure S2

Figure S3

Table S4

Table S5

Table S6

Table S7

Table S8

Table S9

Figure S10

Figure S11

supplemental figure legends and supplemental table titles

## Data Availability

The expression datasets used to support our findings (Supplementary Fig. S2) are available publicly at https://www.ncbi.nlm.nih.gov/geo/query/acc.cgi?acc=GSE21501 and https://icgc.org/icgc/cgp/68/304/798. The expression dataset used for validation is publically accessible on https://portal.gdc.cancer.gov/. Raw data on the patient cohorts employed in this study can be obtained from the corresponding author upon reasonable request.
