# Supplementary figures and images for: TPX2 expression as a negative predictor of gemcitabine efficacy in pancreatic cancer
Source: Br J Cancer. 2023 May 4;129(1):175–82. doi: 10.1038/s41416-023-02295-x (PMC10307892; doi:10.1038/s41416-023-02295-x)

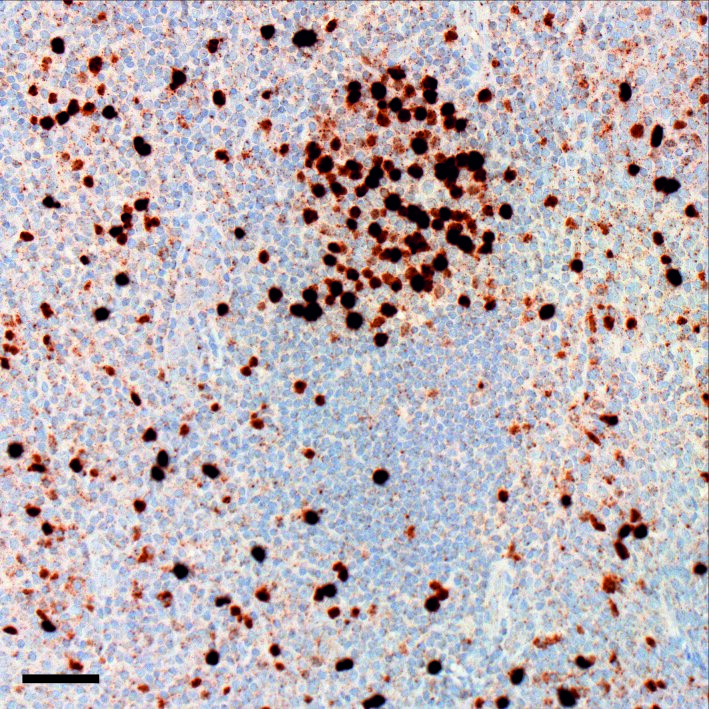

Supplement: Supplementary file 1 — Figure S1 [file 41416_2023_2295_MOESM1_ESM.jpg]

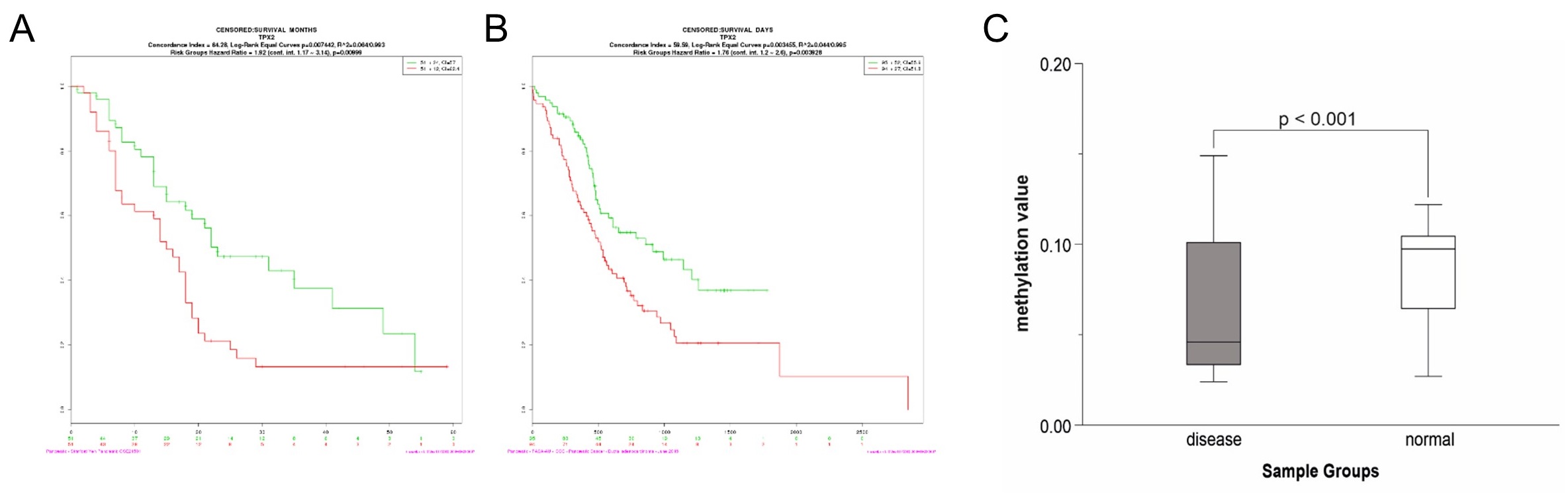

Supplement: Supplementary file 2 — Figure S2 [file 41416_2023_2295_MOESM2_ESM.jpg]

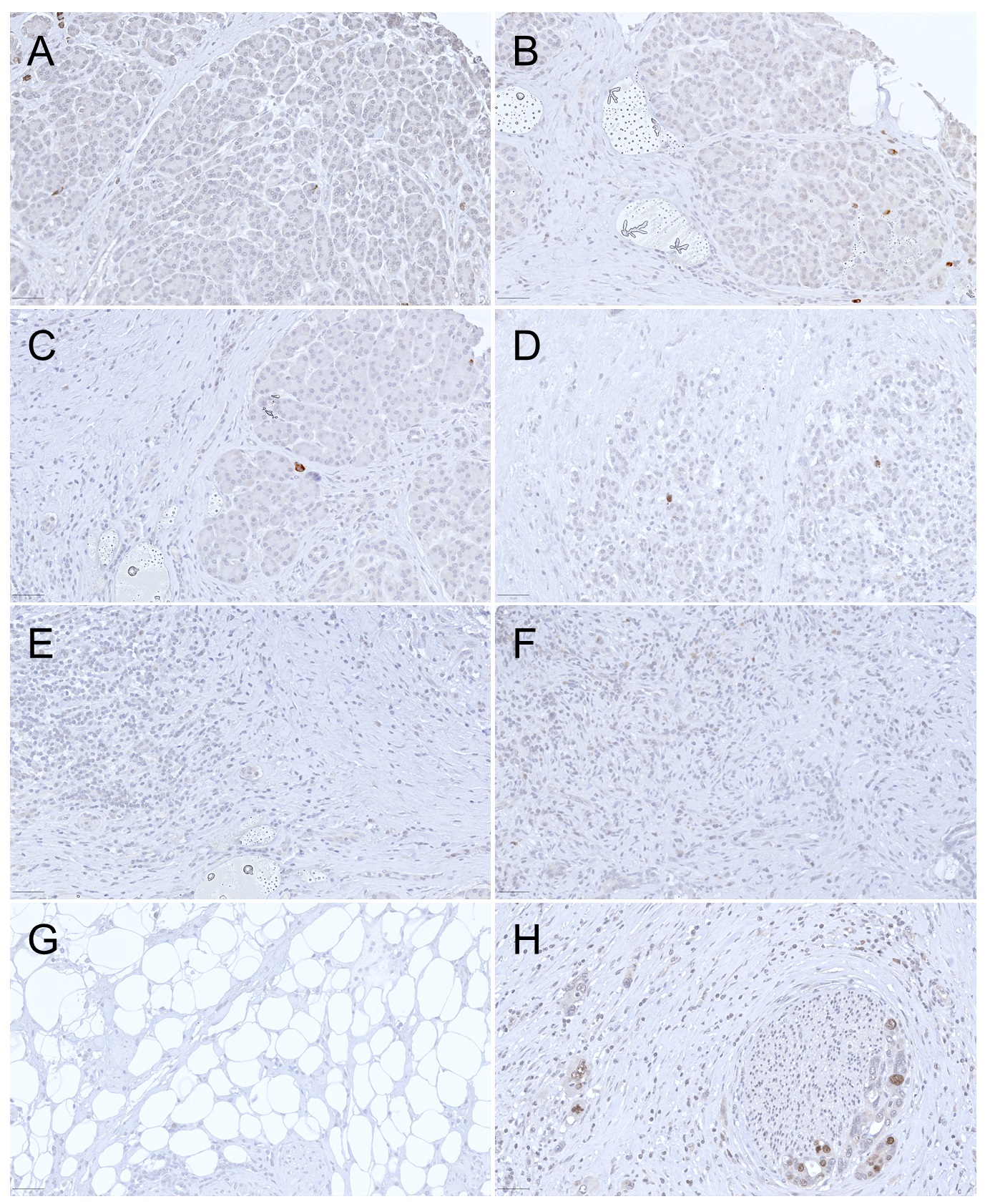

Supplement: Supplementary file 3 — Figure S3 [file 41416_2023_2295_MOESM3_ESM.jpg]

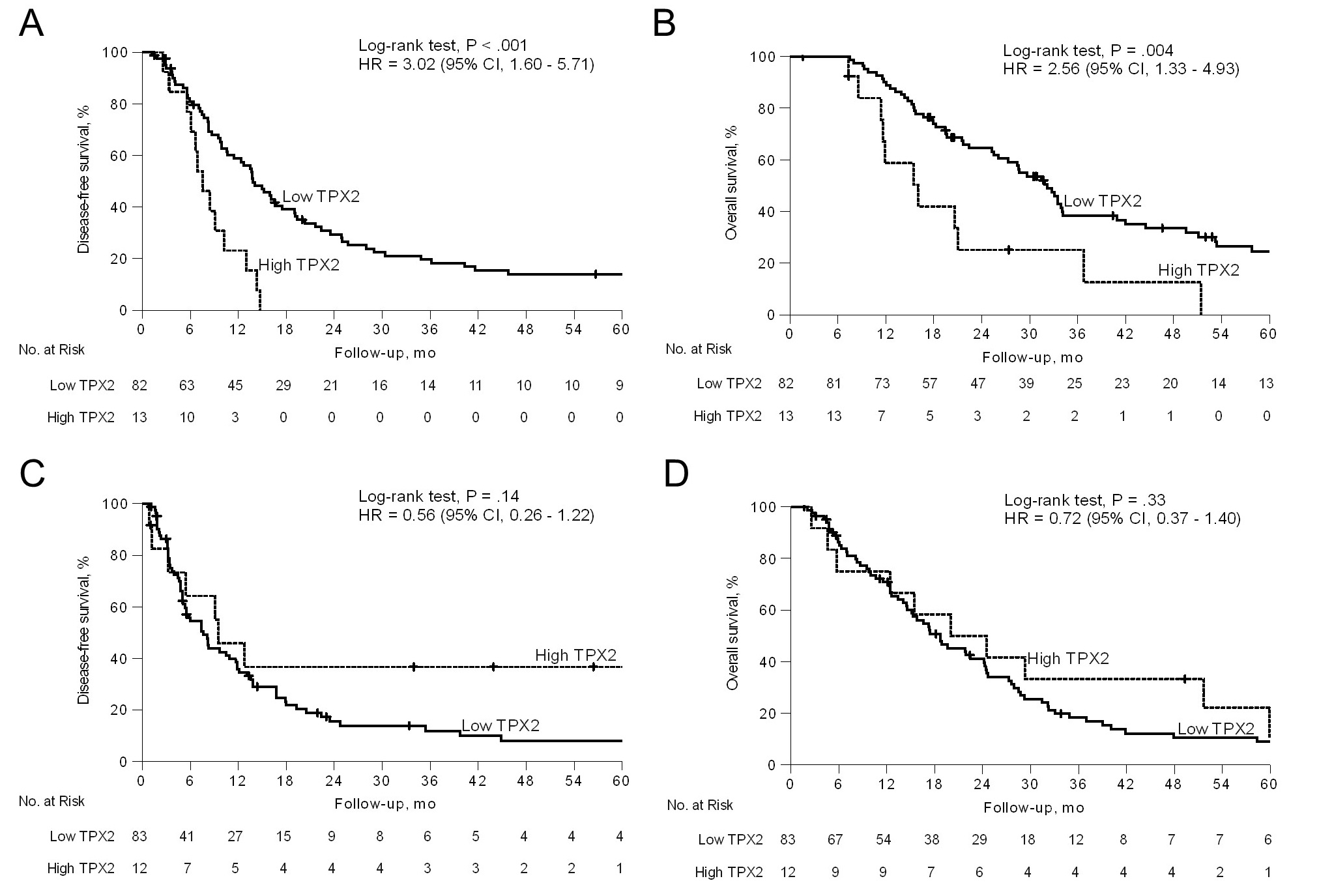

Supplement: Supplementary file 10 — Figure S10 [file 41416_2023_2295_MOESM10_ESM.jpg]

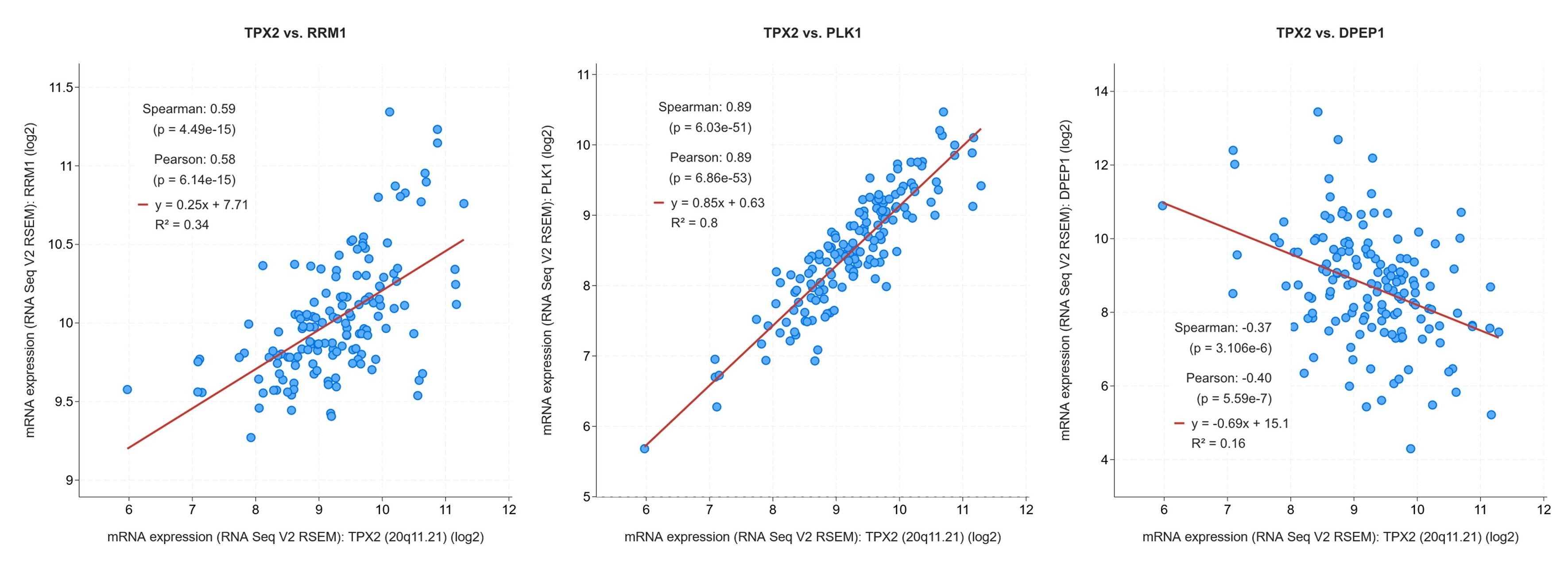

Supplement: Supplementary file 11 — Figure S11 [file 41416_2023_2295_MOESM11_ESM.jpg]
