# Supplementary material for: TPX2 expression as a negative predictor of gemcitabine efficacy in pancreatic cancer
Source: Br J Cancer. 2023 May 4;129(1):175–82. doi: 10.1038/s41416-023-02295-x (PMC10307892; doi:10.1038/s41416-023-02295-x)
Supplement: Supplementary file 12 — supplemental figure legends and supplemental table titles [file 41416_2023_2295_MOESM12_ESM.docx]

**Supplemental figure legends**

**Figure S1**

Immunohistochemical detection of TPX2 expression in normal human tonsil tissue. 200-fold magnification. Scale bar indicates 50 µm.

**Figure S2**

Kaplan-Meier curves and log-rank tests for OS according to TPX2 expression in (A) the GSE21501 dataset, (B) the PACA-AU ICGC dataset. Crossed lines indicate censored cases. Comparison of TPX2 gene methylation levels in PDAC and normal pancreatic tissue according to DiseaseMeth 2.0 database (C).

**Figure S3**

TPX2 expression in non-neoplastic tissue adjacent to invasive PDAC. Immunohistochemical detection of TPX2 in exemplary areas of normal exocrine parenchyma (A,B), exocrine parenchyma with reactive changes and chronic pancreatitis (C,D), chronic pancreatitis (E,F) as well as peripancreatic fat tissue (G) show no cells with strong nuclear TPX2 expression. Occasional plasma cells display unspecific cytoplasmic positivity (B - D). PDAC (partially as perineural invasion), shows strong and specific nuclear expression, whereas the nerves, fibroblasts, and immune cells etc.) remain mostly negative (H). Scale bars indicate 50 µm.

**Figure S10**

*High TPX2 expression is associated with dismal prognosis in resected pancreatic cancer patients treated with adjuvant gemcitabine-based chemotherapy*

Univariate analyses (Kaplan–Meier curves and log-rank tests) in the propensity-score matched cohorts (n=95 each) for DFS and OS in the gemcitabine-based adjuvant treatment cohort (A, B) and the non-gemcitabine-based adjuvant treatment cohort (C, D) according to TPX2 expression.

**Figure S11**

*TPX2 expression strongly correlates with the expression of the gemcitabine-resistance associated genes RRM1 and PLK1 but correlates negatively with the expression of the gemcitabine-sensitivity associated gene DPEP1*

Scatter plots showing the positive association of mRNA expression levels of TPX2 and RRM1 and PLK1 as well as the negative association to DPEP1 in the TCGA firehose legacy dataset (n=179).

**Supplemental table titles**

**Table S4**

Univariate analysis (log-rank test) of the association of clinicopathological patient characteristics to OS and PFS times in the overall advanced PDAC study cohort.

**Table S5**

Univariate analysis (log-rank test) of the association of TPX2 expression to OS and PFS times in the advanced PDAC overall study cohort and each subgroup.

**Table S6**

Multivariate Cox regression analysis of PFS- and OS-associated factors in the advanced pancreatic cancer study cohort.

**Table S7**

Comparison of clinicopathological patient characteristics according to TPX2 expression in the advanced PDAC study cohort.

**Table S8**

Multivariate Cox regression analysis of DFS- and OS-associated factors in the resected PDAC cohort.

**Table S9**

Comparison of clinicopathological patient characteristics according to the applied adjuvant treatment in the resected PDAC study cohort after propensity score matching.
